# Capsaicin reduces Alzheimer-associated tau changes in the hippocampus of type 2 diabetes rats

**DOI:** 10.1371/journal.pone.0172477

**Published:** 2017-02-22

**Authors:** Weijie Xu, Juanhong Liu, Delin Ma, Gang Yuan, Yan Lu, Yan Yang

**Affiliations:** 1 Department of Endocrinology, Tongji Hospital, Tongji Medical College, Huazhong University of Science and Technology, Wuhan, Hubei, China; 2 Department of Endocrinology and Metabolism, Zhongshan Hospital, Fudan University, Shanghai, China; Shanghai Diabetes Institute, CHINA

## Abstract

Type 2 diabetes (T2D) is a high-risk factor for Alzheimer’s disease (AD) due to impaired insulin signaling pathway in brain. Capsaicin is a specific transient receptor potential vanilloid 1 (TRPV1) agonist which was proved to ameliorate insulin resistance. In this study, we investigated whether dietary capsaicin could reduce the risk of AD in T2D. T2D rats were fed with capsaicin-containing high fat (HF) diet for 10 consecutive days (T2D+CAP). Pair-fed T2D rats (T2D+PF) fed with the HF-diet of average dose of T2D+CAP group were included to control for the effects of reduced food intake and body weight. Capsaicin-containing standard chow was also introduced to non-diabetic rats (NC+CAP). Blood glucose and insulin were monitored. The phosphorylation level of tau at individual sites, the activities of phosphatidylinositol 3 kinase/protein kinase B (PI3K/AKT) and glycogen synthase kinase-3β (GSK-3β) were analyzed by Western blots. The results revealed that the levels of phosphorylated tau protein at sites Ser199, Ser202 and Ser396 in hippocampus of T2D+CAP group were decreased significantly, but these phospho-sites in T2D+PF group didn’t show such improvements compared with T2D group. There were almost no changes in non-diabetic rats on capsaicin diet (NC+CAP) compared with the non-diabetic rats with normal chow (NC). Increased activity of PI3K/AKT and decreased activity of GSK-3β were detected in hippocampus of T2D+CAP group compared with T2D group, and these changes did not show in T2D+PF group either. These results demonstrated that dietary capsaicin appears to prevent the hyperphosphorylation of AD-associated tau protein by increasing the activity of PI3K/AKT and inhibiting GSK-3β in hippocampus of T2D rats, which supported that dietary capsaicin might have a potential use for the prevention of AD in T2D.

## Introduction

Alzheimer’s disease(AD) is a devastating neurodegenerative disorder and exhibits two hallmark brain lesions—neurofibrillary tangles and senile plaques [1]. NFTs are formed by intraneuronal accumulation of paired helical filaments composed of abnormally hyperphosphorylated tau protein [2, 3], and senile plaques contain amyloid-β peptide (Aβ). Although the precise significance of these pathological findings remains elusive, more recent estimations pointed out that abnormal hyperphosphorylation of tau protein in hippocampus is critical to the progression of neurodegeneration in AD [4, 5].

Diabetes is one of the most common metabolic diseases worldwide and type 2 diabetes (T2D) accounts for 90–95% of all diabetes. T2D is characterized by peripheral insulin resistance that leads to glucose intolerance and hyperglycemia. Cognitive impairment and dementia have become the new complications of T2D, with the elongation of T2D patients' life expectancy. It’s concluded that T2D patients are at about 60% greater risk for the development of dementia compared with those without diabetes [6]. It was demonstrated that hypoinsulinemia and down-regulated insulin signaling were observed in the brains of T2D rats even there was a hyperinsulinemia in periphery [7–9]. Down-regulated insulin signaling pathway in the brain played a critical role in the development of neurodegenerative disorders, following activated of GSK-3β, an important component of insulin signaling pathway and a key tau kinase in the brain [10, 11]. It was also showed that intranasal insulin administration reversed the reduction of brain insulin signaling and tau hyperphosphorylation in the diabetic rat brains [12], which suggested that T2D might increase the risk for developing AD through the impairment of brain insulin signaling pathway.

Capsaicin is the major pungent ingredient in hot chili peppers and is a highly selective agonist for the transient receptor potential vanilloid 1 (TRPV1) [13]. TRPV1 is a Ca^2+^-permeable nonselective cation channel highly expressed in dorsal root ganglion cells and primary sensory afferents, where it is involved in thermal nociception in addition to the burning sensation we feel upon eating spicy food [14]. TRPV1 has also been identified in the hippocampus, striatum, hypothalamus, and cerebellum in the brain of human and rat’s [15]. Growing evidences supported the idea that capsaicin consumption facilitated body weight management [16] and TRPV1 had a potential therapeutic value for obesity and diabetes [17]. In the central nervous system, it was suggested that activation of TRPV1 could enhance synaptic transmission, synaptic plasticity, and hippocampal memory formation [18]. A recent study revealed that capsaicin had an effect of prevention on stress-induced AD-like changes in brain of rats [19]. However, whether and how dietary capsaicin could prevent AD or AD-like changes in brain have yet to be elucidated.

We hypothesized that dietary capsaicin might have a beneficial effect on preventing AD or AD-like changes in the hippocampus of T2D rats. Therefore, the T2D rats model by feeding with high fat diet and a single intraperitoneal injection of streptozotocin (STZ) were used. The phospho-sites of tau protein, the activities of PI3K/AKT and GSK-3β were examined after dietary capsaicin exposure, in order to elucidate whether PI3K/AKT plays a critical role in the regulating effect of dietary capsaicin on tau phosphorylation.

## Materials and methods

### Animals

A total of 60 male Sprague-Dawley rats, aged between 10 and 12 weeks, weighing 200–250 g, were purchased from the Animal Center of Tongji Medical College, Huazhong University of Science and Technology (Wuhan, China). Animals were individually housed on a light/dark cycle 12h/12h (lights on at 21:00h) in a temperature (23°C) and humidity (50 ± 10%) controlled room. All procedures were approved by the Animal Care and Use Committee of the Tongji Medical College, Huazhong University of Science and Technology, Wuhan, China.

### Experiment design

**Fig 1:**
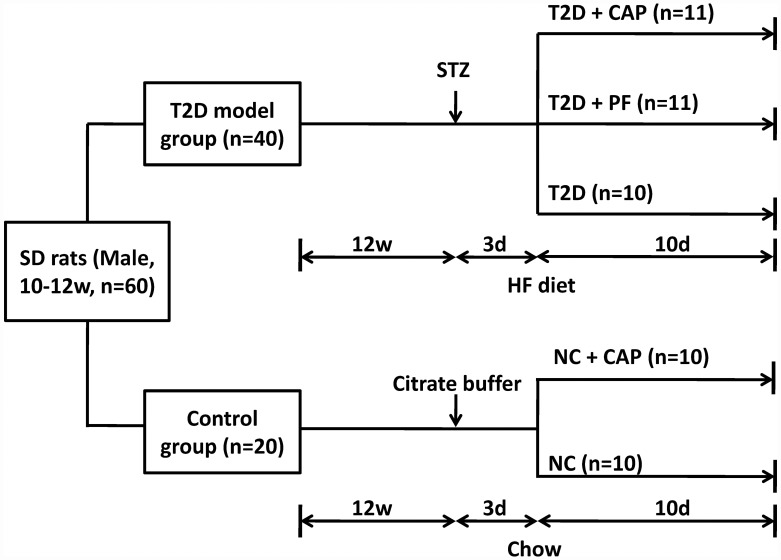
The diagram for experimental design. 60 male SD rats were included in this study. 40 rats were assigned to T2D model group and fed by HF diet, while 20 rats were assigned to control group and fed by standard chow. 12 weeks later, rats in T2D model group were injected with STZ, while rats in the control group were injected with citrate buffer only. The whole blood glucose and plasma insulin were monitored 3 days after the STZ or citrate buffer injection. Hyperglycemia was verified 3 days after the STZ injection, 8 rats did not develop diabetes within 3 days after the STZ injection were excluded from further assessment. Thereafter, capsaicin-containing HF diet was introduced to T2D+CAP group (n = 11), rats in T2D+PF (n = 11) group were pair-fed with HF diet according to the amount of HF diet intake of T2D+CAP group one day before, and T2D group (n = 10) got free access to HF diet. For control group, half of the rats were introduced to capsaicin-containing standard chow (NC+CAP, n = 10) while another half (NC, n = 10) still got free access to standard chow. After 10 days of capsaicin exposure, whole blood glucose and plasma insulin were monitored again before sacrifice.

The procedures of this experiment are shown in Fig 1. After one week of habituation, 40 rats were randomly assigned to T2D model group and fed by 45% high-fat diet (4.7 kcal/g; Research Diets, Inc, New Brunswick, NJ, D12451; 45% fat, 20% protein, and 35% carbohydrate), while 20 rats were assigned to control group fed by standard chow (SC, 3.1 kcal/g; Harlan Laboratories, Inc.Teklad Global 18% Protein Rodent Diet; 18% fat, 24% protein, and 58% carbohydrate). All rats got free access to food and water. After 12 weeks feeding by their assigned diet, rats in T2D group were intraperitoneal injected with STZ (Sigma, USA) once with a dose of 35 mg/kg, which is a commonly used approach to develop T2D rat model [20]. At the same time, rats in the control group were injected with citrate buffer only.

Food was taken away during 9:00h-21:00h (dark cycle) and the blood glucose and plasma insulin were monitored at 20:00h before the light cycle. After examination, the food was put back and the light cycle began. The blood sample was considered to be fasting status. Rats were considered hyperglycemic if the fasting blood glucose level was higher than 16 mmol/L (normal range: 5–8 mmol/L). The rats with HF diet and STZ injection did not develop hyperglycemia within three days after the STZ injection were excluded from further study. Thus, the final numbers of T2D rats were 32.

Three days after the intraperitoneal injection, rats were further randomly grouped. T2D rats were divided into three groups: T2D+CAP group, T2D+PF group, and T2D group. Rats in T2D+CAP group were fed with 0.01% capsaicin [21–23] containing 45% HF diet (capsaicin was purchased from Wako, Osaka, Japan, 0.01% capsaicin containing 45% HF diet was made in Wuhan Goodbio Technology CO., LTD). The daily amount of HF diet given to the rats in T2D+PF group was the average amount of HF diet intake of T2D+CAP group one day before. Rats in T2D group still got free access to HF diet. For the control group (non-diabetic rats), half of the rats were randomly assigned to NC+CAP group and got 0.01% capsaicin containing standard chow (0.01% capsaicin containing standard chow diet was made in Wuhan Goodbio Technology CO.,LTD), while another half were in NC group and still got free access to standard chow. All rats received water ad libitum. Food intake was measured one hour after light onset daily. Daily energy intake was calculated as energy of diet (3.1 kcal/g of standard chow and capsaicin-containing standard chow, 4.7 kcal/g of 45% high-fat diet and capsaicin-containing 45% high-fat diet) multiplied by daily grams of diet consumed. All the procedures carried out in this study were included in Fig 1.

### Measurement of plasma glucose, plasma insulin and HOMA-IR

Blood glucose levels were measured by using a 3-μl drop of blood obtained by nicking the tail blood vessel and a One Touch Ultra Link Blood Glucose Meter (LifeScan, China). Plasma insulin was determined by sandwich enzyme-linked immunosorbent assay (ELISA), according to the manufacturer's instruction (REI034, Bogoo Co., Shanghai, China). Insulin resistance was assessed by homeostasis model assessment-insulin resistance (HOMA-IR) using the formula of HOMA-IR = fasting insulin (mIU/L) × fasting blood glucose (mmol/L) / 22.5 [24].

### Tissue preparation

Just before the light cycle (21:00h), rats were sacrificed by decapitation without anesthesia, and the brains were rapidly removed, the hippocampus were dissected on an ice-cooled board and stored at -80°C for Western blotting analysis.

### Western blotting

Brain tissue samples were homogenized in a buffer containing 40 mM Tris-HCl (pH 7.0), 1% Triton X-100, 0.2% SDS, 1.0 mM sodium deoxycholate, 1.0 mM EGTA, 1.0 mM EDTA, 1.0 mM Na_3_VO_4_, 50 mM NaF, 1.0 mM PMSF, and 2.0 μg/ml each of aprotinin, leupeptin and pepstatin. The homogenates were centrifuged at 16,000 rpm for 15 min, and the supernatants were stored in aliquots at -80°C until use. The tissue extracts were separated in SDS-polyacrylamide gels and then transferred onto polyvinylidene difluoride membrane (Millipore, CA). Nonspecific binding sites on the membrane were blocked by 5% bovine serum albumin in 0.1% TBS/Tween-20 (TBST) for 1 h. The membrane was then incubated with specific primary antibodies (Table 1) overnight at 4°C. After washing with TBST, the membrane was incubated with horseradish peroxidase-conjugated secondary antibody (1:2000; Cell signaling Technology, Beverly, MA) for 2 hours at room temperature. Immunoreactive bands were visualized by using the Super Signal West Pico Chemi-luminescent Substrate (Pierce Biotechnology, Rockford, IL) and Chem Doc XRS with Quantity One software (BioRad, USA). The protein bands were scanned and the intensities of the bands were measured using Image-pro Plus 6.0 analysis software (Media Cybernetics, Rockville, MD, USA).

**Table 1:** Primary antibodies employed in this study.

Antibody	Type^a^	Specificity ^b^	Source	Cat. #
Tau-5	Mono	Total tau	Abcam, Cambridge, MA, USA	ab80579
pS199	Mono	P-tau at Ser199	Abcam	ab81268
pS202	Mono	P-tau at Ser202	Abcam	ab108387
pS396	Poly	P-tau at Ser396	Abcam	ab192208
Tau-1	Mono	Dephosphorylated at Ser198/Ser199/Ser202	Millipore, Billerica, MA, USA	MAB3420
GSK-3β	Mono	Total GSK-3β	Cell signaling, Technology, Beverly, MA, USA	9315
p-GSK-3β	Poly	p-GSK-3β at Ser9	Cell signaling	9336
AKT	Poly	Total AKT	Abcam	ab8805
p-AKT	Poly	p-AKT at Thr308	Abcam	ab38449
Actin	Poly	β-actin	Santa Cruz, Santa Cruz, CA, USA	sc-1616-R

^a^ Poly, polyclonal; Mono, monoclonal.

^b^P-tau, phosphorylated tau.

### Statistical analysis

Data were analyzed by ANOVA, followed by *post hoc* test, or Student’s *t* test using the program GraphPad Prism 5.0 (GraphPad Prism Software Inc., San Diego, USA). Results are expressed as mean ± SD, and *P* < 0.05 was considered statistically significant.

## Results

### Dietary capsaicin ameliorates hyperglycemia and insulin resistance of type 2 diabetic rats

**Fig 2:**
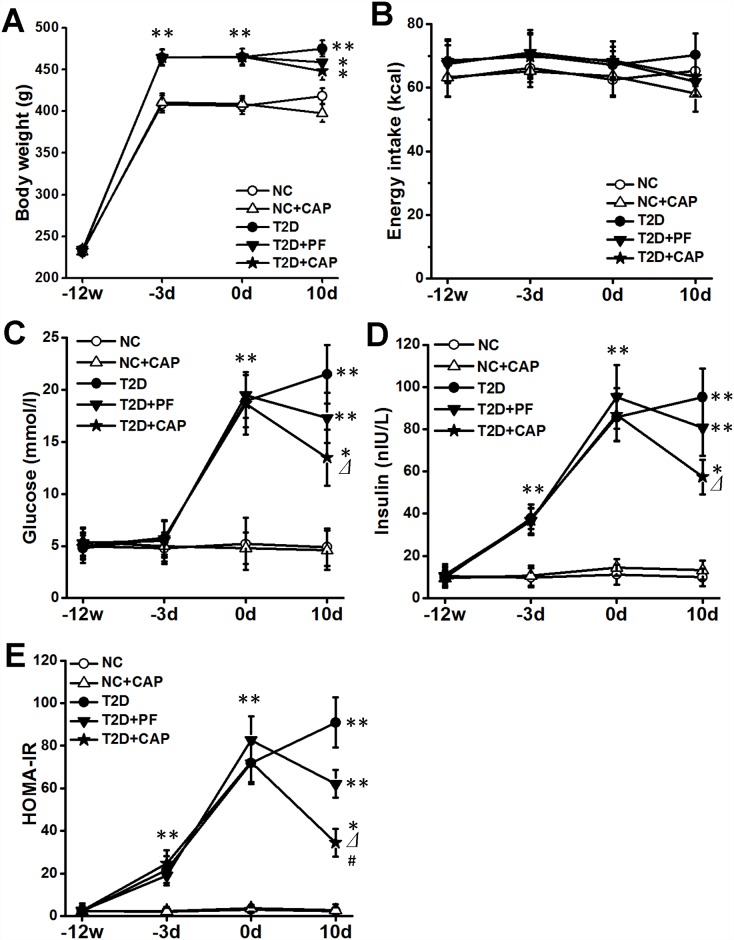
Changes of body weight (2A), energy intake (2B), blood glucose (2C), plasma insulin (2D) and HOMA-IR (2E). Data are mean ± SD. Differences *vs*. NC group are indicated as *P < 0.*05*, **P < 0.*01*; differences *vs*. T2D group are indicated as ⊿*P < 0*.*05* and differences *vs*. T2D+PF group are indicated as #*P < 0*.*05*. ‘NC’ indicated non-diabetic rats fed with standard chow, ‘NC+CAP’ indicated non-diabetic rats fed with capsaicin-containing standard chow, ‘T2D’ indicated T2D rats with free access to HF diet, ‘T2D+PF’ indicated T2D rats pair-fed with HF diet according to the amount of HF diet intake of T2D+CAP group one day before. ‘T2D+CAP’ indicated T2D rats fed with capsaicin-containing HF diet. Measurements were taken on week -12, day -3, day 0 and day 10.

Within 3 days after the STZ injection, 32 of the 40 HF-diet fed rats (80%) developed T2D, as assessed by blood glucose in fasting status (>16 mmol/L) (Fig 2C). The remaining 8 rats that did not meet the criteria for T2D were then excluded from further study. As expected, the HF-fed rats gained their body weights at higher rates than the control group rats (Fig 2A). STZ injection did not affect their body weight, but increased the fasting blood glucose level from about 5.0 mmol/L to about 18 mmol/L within 3 days. Also, the plasma insulin and HOMA-IR increased as markedly as blood glucose level did after STZ injection. The results confirmed the success of modeling of type 2 diabetes in rats fed with HF diet and got STZ injection.

During the 10-day of capsaicin-containing diet regiment, rats in capsaicin groups consumed less energy than did rats in the same diet condition. However, there was no significant difference of energy intake among all five groups on the 10^th^ day of capsaicin exposure (Fig 2B). After 10 days of capsaicin exposure, rats in all T2D groups still weighted significantly more than non-diabetic groups (Fig 2A). Although T2D+CAP rats weighed less than the T2D group, the difference did not reach a significant level. Also, there was no significant reduction of body weight in NC+CAP group comparing to NC group.

When compared with T2D group, capsaicin produced significant reductions in blood glucose, plasma insulin and HOMA-IR after 10 days of administration in T2D+PF group. We used a T2D pair-fed group (T2D+PF group) to control for the effects of reduced food intake and body weight. In our study, there were no significant improvements of blood glucose, plasma insulin and HOMA-IR between T2D and T2D+PF groups after capsaicin exposure, which indicated direct beneficial effects of capsaicin on glucose metabolism and insulin resistance. No significant changes of blood glucose, plasma insulin and HOMA-IR between NC+CAP and NC groups after capsaicin exposure, which suggested no effects of capsaicin on glucose metabolism and insulin resistance on normal diet condition.

### Dietary capsaicin reduced hyperphosphorylation of tau protein in the hippocampus

**Fig 3:**
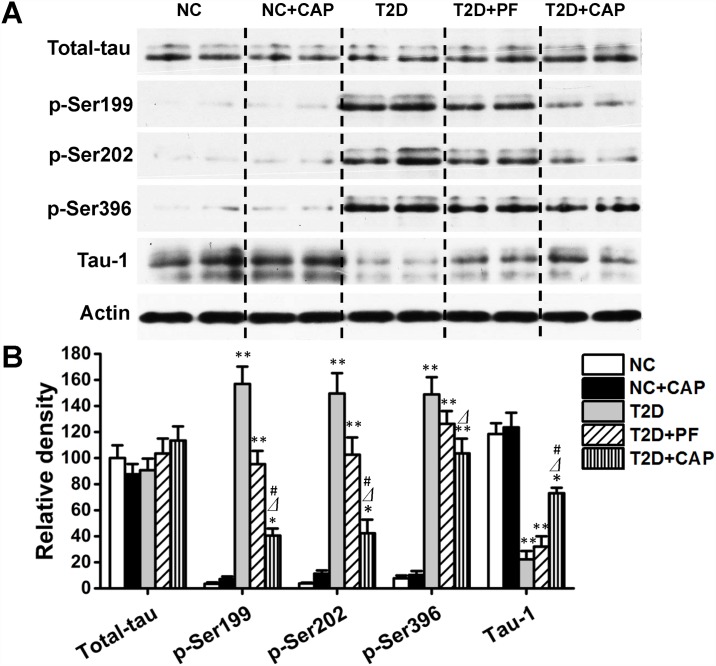
Western blotting analysis of phosphorylation of tau protein in rat hippocampus. 3A, western blots of the crude hippocampal extracts (7–13 μg/lane) were analyzed with several site-specific tau antibodies to detect the phosphorylation levels of tau at the specific sites. Each lane was from an individual rat. Actin blot was included as a loading control. 3B, the blots as shown in panel 3A were quantitated desitometrically and for quantitation of tau phosphorylation level at each site, data had been normalized by the level of total tau. All data are presented as mean ± SD of the relative immunoreactivities. *P < 0.*05*,**P < 0.*01* as compared to NC group; ⊿*P < 0*.*05* as compared to T2D group and #*P < 0*.*05* as compared to T2D+PF group.

It has been reported that an increasing in tau hyperphosphorylation at several AD-associated hyperphosphorylation sites in the brains of type 2 diabetic patients [10] and rodents [12]. The sites of tau protein we tested are the mostly studied tau hyperphosphorylation sites in AD. In the present study, there was almost no difference in total tau protein level among these 5 groups. We also observed obvious higher level of tau phosphorylation at Ser199, Ser202 and Ser396 in the hippocampus of T2D group than other groups (Fig 3). Tau-1 is a monoclonal tau antibody that recognizes non-phosphorylated sites of Ser198/Ser199/Ser202 on tau protein. In this study, a markedly reduction of tau-1 was observed in T2D group compared to NC group which confirmed that hyperphosphorylation of tau at these sites. Our results are consistent with our previous studies [8, 9]. In our hypothesis, capsaicin might prevent the hyperphosphorylation of tau protein in hippocampus of T2D rats. In the present study, we found that capsaicin significantly prevent tau phosphorylation at all three phosphorylation sites in T2D+CAP group compared to T2D group, and the level of tau-1 in T2D+CAP was significantly lower. However, in T2D+PF group, tau phosphorylation at these sites was not significantly lower than T2D group. These results indicated that dietary capsaicin inhibits AD-associated tau hyperphosphorylation in the T2D brains.

### Restoration of brain AKT and GSK-3β activity in T2D rats by dietary capsaicin

AKT and GSK-3β are important protein kinases in the insulin signaling pathway in brain [25]. Upon binding of insulin to its receptor, the insulin signaling pathway is activated; leading to activation of AKT through its phosphorylation at Thr308 [25]. Activated AKT then phosphorylates GSK-3β at Ser9 and inhibits its kinase activity [26]. GSK-3β is also the major tau kinase in the brain and phosphorylates tau at many hyperphosphorylation sites including Ser199 and Ser202 [27]. Phosphorylation of tau on Ser396 was suggested to be a key step in the development of neurofibrillary pathology in Alzheimer’s disease brain [28].

**Fig 4:**
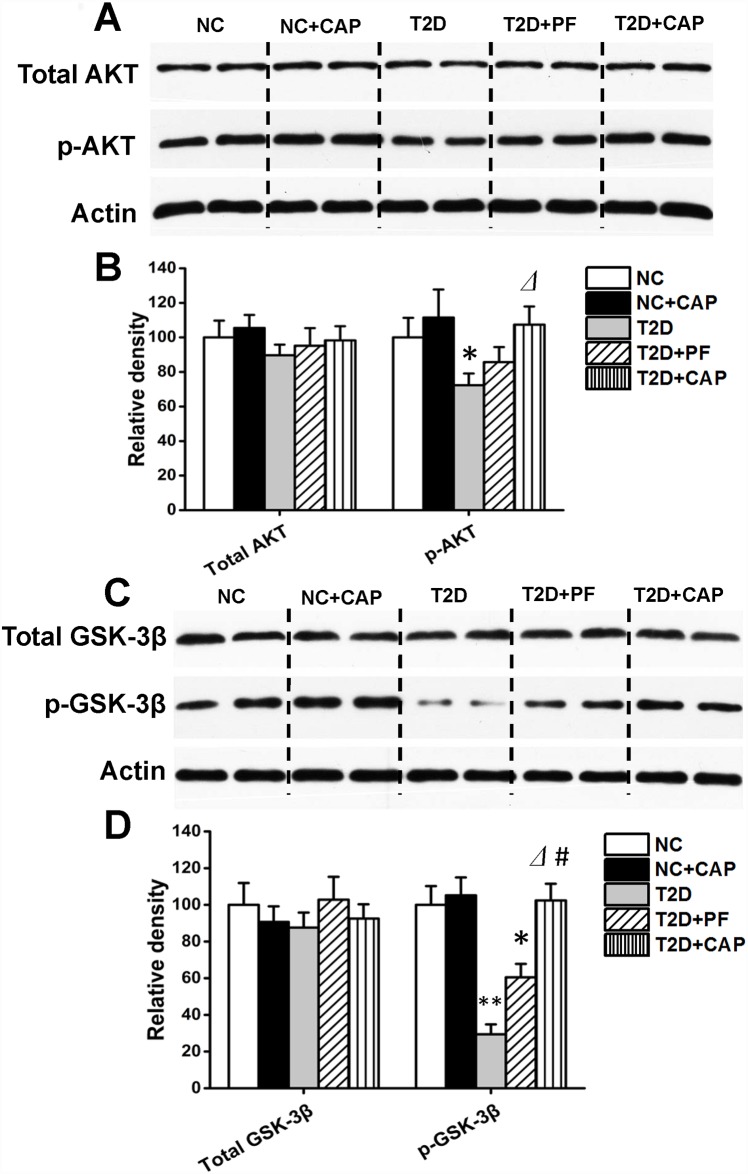
Western blot analysis of insulin signal transduction in rat hippocampus. Crude hippocampal extracts (5–15 μg/lane) were analyzed by Western blots developed with antibodies to total AKT and phosph-AKT (4A, B), total GSK3β and phosph-GSK3β (4C, D). Actin blot was included as a loading control. Each lane was from an individual rat (4A, C), the blots as shown in panel 4B, D were quantitated desitometrically and for quantitation of total AKT and total GSK-3β. All data are presented as mean ± SD of the relative immunoreactivities. *P < 0.*05*,**P < 0.*01* as compared to NC group; ⊿*P < 0*.*05* as compared to T2D group and #*P < 0*.*05* as compared to T2D+PF group.

When we compared these two kinases between NC and T2D rats, we found that phosphorylation of both AKT and GSK-3β was significantly lower in T2D rat brains than NC group, whereas the total levels of these two kinases were unchanged (Fig 4). These results indicated impaired brain insulin signaling that lead to inhibition of AKT and consequently over-activation of GSK-3β. In T2D+CAP group, we found significantly higher level of phosphorylation of both AKT and GSK-3β compared to T2D group. However, in T2D+PF group, level of phosphorylation of AKT and GSK-3β were not significantly higher than T2D group. There were no notable differences of the phosphorylation level of AKT and GSK-3β between NC and NC+CAP group, which suggested almost no effects of capsaicin on phosphorylation of AKT and GSK-3β under normal diet condition. Collectively, these data support a potential role of dietary capsaicin in brain insulin signaling pathway.

## Discussion

During the last three decades, tremendous progress was made in our understanding of the mechanisms of AD. However, discovery of new pharmacological approaches, especially of disease-modifying drugs, has not been successful to date [29]. Lifestyle intervention is the primary strategy for preventing both type 2 diabetes [30] and Alzheimer’s disease [31]. Capsaicin, a natural food ingredient which produces a burning sensation on oral cavity, has less side-effects and is more cost-effective than pharmacological approaches. It is proved that capsaicin ingested with food can be rapidly absorbed by a nonactive process in the gastrointestinal tract and the total absorption capacity is between 50–90% [32]. After being tranported into the portal vein and then into the whole body of both human and rodent, about 5% of unchanged capsaicin crosses the blood brain barrier and goes into the brain tissue [21, 32]. In this present study, we found that short term intervention of dietary capsaicin, a highly selective agonist for TRPV1, increased insulin signaling pathway in hippocampus, which leads to inhibition of GSK-3β, prevention of hyperphosphorylation of tau protein and improved glucose metabolism and insulin resistance without affecting body weight in a rat model of type 2 diabetes. These findings provide novel insights into the possible mechanism by which lifestyle intervention of dietary capsaicin may be useful for preventing AD in type 2 diabetic patients.

Evidences supported that capsaicin was indicated in effects of obesity and improving glucose homeostasis in type 2 diabetes. Dietary capsaicin improved glucose homeostasis and insulin sensitivity, reduced body weight, attenuated inflammation of adipose tissue and liver, enhanced fatty oxidation in high-fat diet fed obese mice [33] and improved visceral fat remodeling [22]. Capsaicin stimulated GLP-1 secretion [23], and TRPV1 expressed in islet β cells could modulate insulin secretion via a mechanism of increased calcium influx [34]. TRPV1 knock-out mice became more obese, insulin resistant and leptin resistant when fed with HF diet [35]. In human short term studies, capsaicin increased satiety, reduced energy and fat intake [36], and stimulated thermogenesis [37], which were likely via stimulation of the sympathoadrenal system [16]. In the present study, we observed that capsaicin improved glucose metabolism and insulin sensitivity in T2D rats, but not non-diabetic rats fed with normal diet. Capsaicin produces a burning sensation in oral cavity, so one may question that capsaicin could decrease the palatability of the rodent diet in our study, so rats fed with capsaicin-containing diet would consume less food. Indeed, energy restriction helps to maintain healthier bodyweight and attenuate T2D [38]. So we introduced T2D+PF group to control for the effects of reduced food intake and body weight. In our study, energy intake of rats in T2D+CAP group robustly decreased on the first few days compared to T2D group, but restored gradually later, and was not significantly different on the 10^th^ day of capsaicin exposure. After capsaicin exposure, T2D+CAP rats showed improved glucose metabolism compared to T2D rats but rats in T2D+PF group didn’t get such improvement compared to T2D group, which indicated that improvements in glucose metabolism and insulin sensitivity were directly the result of capsaicin itself rather than energy intake reduction. However, no significant body weight decrease was seen in T2D+CAP group or T2D+PF group in the end of 10-day exposure of capsaicin compared to T2D group, which may due to the short term exposure of capsaicin.

There is no cure for AD at the present time. Although amyloid-β peptide is the primary trigger of AD according to the amyloid cascade hypothesis [39], neurofibrillary degeneration of abnormally hyperphosphorylated tau is apparently required for the clinical expression of this disease [40]. Abnormal hyperphosphorylation of tau, which is crucial to neurodegeneration, received much attention as an AD drug target recently [41]. In the present study, we found that the levels of some AD-associated tau protein sites (Ser199, Ser202 and Ser396) were hyperphosphorylated in the hippocampus of T2D rats, which is consistent with previous study [8]. After ten days feeding of capsaicin-containing HF diet, significant reduction of hyperphosphorylation of tau protein in the brain of T2D rats was detected on these sites. These results demonstrated that AD-like changes were shown in T2D brain, and dietary capsaicin prevented these AD-like changes. However, NC+CAP rats fed with capsaicin-containing standard chow didn’t show significant changes of tau protein phosphorylation at these sites compared to NC group, which indicated that capsaicin didn’t affect the process of tau protein phosphorylation in non-diabetic and normal diet situation.

Impairment of brain insulin signaling has been implied in the development of AD [10, 42]. As a result, restoring brain insulin signaling is potentially a novel strategy for treating AD [42]. In our present study, deficiency of brain impaired insulin signaling pathway was found in the brain of T2D rats, which is consistent with our previous findings that showing impaired brain insulin signaling pathway in individuals with type 2 diabetes [9, 12]. Proteins involved in insulin signaling pathway was up-regulated in T2D+CAP group compared to T2D rats, which is also consistent with a recent study showed that the administration of extracts of red peppers restored insulin signaling pathway and prevented tau hyperphosphorylation and β-amyloid accumulation in a rat model of T2D combined with β-amyloid-induced dementia [43]. Studies suggested that calcium influx triggered PI3K/AKT signaling pathway in several tissues [44–46]. In our study, whether TRPV1 activation by dietary capsaicin restored PI3K/AKT signaling pathway in hippocampus of T2D rats through a direct Ca^2+^/PI3K/AKT signaling is still unclear. Further studies are needed to demonstrate the specific molecular mechanism.

In conclusion, this study supports the view that dietary capsaicin could partly restore the impaired insulin signaling pathway in brain, whereas ameliorate AD-associated hyperphosphorylated tau protein in T2D. No impairment in brain insulin signaling pathway was seen when capsaicin applied to non-diabetic rats fed with standard chow diet. Therefore, the present study provides possible supporting evidence for the role of dietary capsaicin reducing the risk of AD in T2D.

## Supporting information

S1 Fig Western blot analysis. Western blot analysis of tau protein, AKT, GSK3β in rat hippocampus.
(PDF)

S1 Table Body weight changes. Body weight changes of rats in different groups on week -12, day -3, day 0 and day 10.
(PDF)

S2 Table Energy intake. Energy intake of rats in different groups on week -12, day -3, day 0 and day 10.
(PDF)

S3 Table Fasting blood glucose and fasting plasma insulin levels. Fasting blood glucose and fasting plasma insulin levels of rats in different groups on week -12, day -3, day 0 and day 10.
(PDF)
