# Supplementary figures and images for: Capsaicin reduces Alzheimer-associated tau changes in the hippocampus of type 2 diabetes rats
Source: PLoS One. 2017 Feb 22;12(2):e0172477. doi: 10.1371/journal.pone.0172477 (PMC5321461; doi:10.1371/journal.pone.0172477)

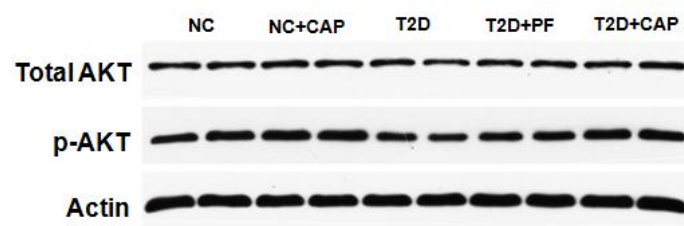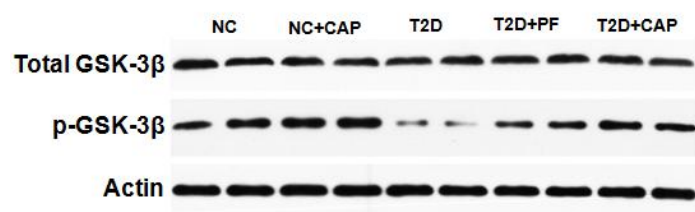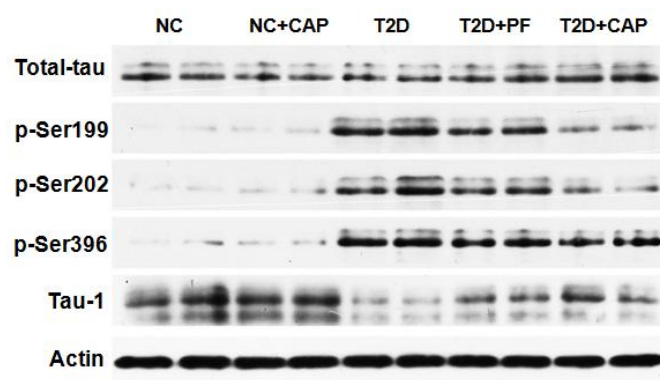

Supplement: S1 Fig — Western blot analysis of tau protein, AKT, GSK3β in rat hippocampus. (PDF) [file pone.0172477.s001.pdf]
