# Supplementary material for: Capsaicin reduces Alzheimer-associated tau changes in the hippocampus of type 2 diabetes rats
Source: PLoS One. 2017 Feb 22;12(2):e0172477. doi: 10.1371/journal.pone.0172477 (PMC5321461; doi:10.1371/journal.pone.0172477)
Supplement: S1 Table — Body weight changes of rats in different groups on week -12, day -3, day 0 and day 10. (PDF) [file pone.0172477.s002.pdf]

| Body weight，g | -12w | -3d | 0d | 10d |
|---|---|---|---|---|
| NC | 235.6 | 412.5 | 415.2 | 430.3 |
| | 238.7 | 392.1 | 389.5 | 406.3 |
| | 225.7 | 409.5 | 406.3 | 418.3 |
| | 242.1 | 400 | 400.5 | 415 |
| | 237.4 | 413.5 | 409.8 | 417.6 |
| | 233.6 | 423.5 | 418.9 | 427.9 |
| | 230.7 | 398.7 | 396.7 | 406.1 |
| | 235.6 | 408.5 | 406.7 | 420.4 |
| | 243 | 420.5 | 420.3 | 432.1 |
| | 229.8 | 396.2 | 392.1 | 405.3 |
| | 224.1 | 415.4 | 412.5 | 418.6 |
| | | | | |
| NC+CAP | 236.1 | 424.5 | 423.1 | 418.5 |
| | 220.5 | 397.9 | 402 | 397.8 |
| | 227.8 | 405.6 | 399.7 | 382.4 |
| | 234.6 | 412.3 | 410.4 | 399.3 |
| | 239.5 | 415 | 410.3 | 403.1 |
| | 226.5 | 395.2 | 393.4 | 385.6 |
| | 235.7 | 425.6 | 420.1 | 402.1 |
| | 231.6 | 410.5 | 408 | 389.1 |
| | 237.4 | 415.3 | 415 | 403.2 |
| | 229.5 | 409.1 | 408.7 | 400.3 |
| | 231.4 | 410.2 | 407.3 | 397.1 |
| | | | | |
| T2D | 234.1 | 470.5 | 468.1 | 475.6 |
| | 236 | 465.3 | 460.1 | 468.7 |
| | 229.8 | 451.6 | 456.3 | 467.9 |
| | 241.2 | 470.5 | 468 | 479.8 |
| | 239.4 | 480.3 | 485.6 | 492.6 |
| | 238.5 | 472.7 | 475.3 | 487.5 |
| | 228.1 | 457.6 | 459.7 | 468 |
| | 229.3 | 461.2 | 462.8 | 468.5 |
| | 234.5 | 464 | 462.3 | 470.1 |
| | 231.7 | 459.3 | 457.3 | 463.2 |
| | | | | |
| T2D+PF | 229.1 | 464.3 | 466.2 | 462.1 |
| | 227.3 | 459.2 | 458.4 | 450.2 |
| | 235.6 | 482.3 | 483.1 | 475.2 |
| | 238.9 | 470.2 | 465.3 | 463.8 |
| | 234.5 | 457.8 | 458.9 | 456.7 |
| | 238.1 | 462.8 | 464.2 | 461.9 |
| | 230.6 | 460.5 | 457.9 | 460.2 |
| | 232.5 | 450.2 | 445.9 | 447.8 |
| | 238.5 | 472.3 | 478.5 | 481.3 |

| | | | | |
|---|---|---|---|---|
| | 240.3 | 469.5 | 465.6 | 465.8 |
| T2D+CAP | 233.5 | 467.2 | 472.8 | 453.1 |
| | 245.1 | 475.3 | 478.3 | 460.3 |
| | 232.7 | 468.3 | 467.9 | 457.2 |
| | 228.5 | 459.7 | 461.2 | 445.3 |
| | 229.3 | 462.5 | 461.7 | 441.7 |
| | 235.3 | 485.3 | 482.9 | 460.4 |
| | 229.8 | 450.8 | 445.2 | 430.7 |
| | 232.4 | 462.3 | 464.8 | 439.2 |
| | 227.5 | 458.2 | 459.3 | 445.7 |
| | 234.2 | 462.1 | 464.7 | 452 |
