# Supplementary material for: Capsaicin reduces Alzheimer-associated tau changes in the hippocampus of type 2 diabetes rats
Source: PLoS One. 2017 Feb 22;12(2):e0172477. doi: 10.1371/journal.pone.0172477 (PMC5321461; doi:10.1371/journal.pone.0172477)
Supplement: S2 Table — Energy intake of rats in different groups on week -12, day -3, day 0 and day 10. (PDF) [file pone.0172477.s003.pdf]

Energy intake，kcal	-12w	-3d	0d	10d
NC	67.902	72.336	68.724	74.382
	52.614	58.234	62.168	51.357
	68.372	65.812	70.253	75.45
	59.373	68.902	60.574	66.782
	65.268	59.843	73.632	67.975
	71.525	77.528	62.467	66.4
	60.751	62.662	56.9	66.373
	55.351	68.316	52.672	60.109
	64.239	64.235	67.882	55.327
	56.781	60.442	57.157	65.863
	72.173	65.857	59.235	68.167
NC+CAP	65.782	70.571	62.356	57.319
	54.395	65.436	54.881	58.747
	55.469	55.781	65.747	66.896
	71.423	78.099	78.045	66.942
	62.361	53.449	52.365	45.691
	53.449	68.671	58.367	56.382
	71.136	66.782	68.585	64.72
	70.832	63.855	62.922	52.955
	61.5	57.233	65.345	52.976
	73.1	70.672	58.179	63.274
	62.49	65.221	72.614	60.721
T2D	61.963	70.747	78.248	68.585
	67.166	71.382	65.652	69.125
	65.639	73.274	70.004	79.099
	78.991	64.801	68.423	88.559
	58.598	65.449	66.436	62.436
	75.68	81.503	72.369	64.801
	70.423	72.477	56.192	60.179
	63.382	52.557	60.475	65.462
	71.423	72.773	73.315	70.477
	72.682	74.261	68.505	77.099
T2D+PF	76.223	72.682	65.138	63
	71.153	69.531	66.693	63.4
	58.719	68.112	64.395	62.9
	74.194	56.287	60.544	62.5
	68.477	61.963	65.084	63
	73.883	77.613	81.248	62.8
	65.451	78.086	70.721	63.2
	58.503	66.22	70.004	62.4
	59.125	78.991	72.328	63

	75.68	82.086	70.369	62.7
T2D+CAP	68.747	75.248	66.585	65.382
	72.382	58.652	69.125	64.395
	68.274	70.004	81.099	66.693
	64.801	71.423	78.559	70.95
	63.449	81.436	62.436	66.004
	75.477	75.369	63.801	59.125
	55.503	63.192	58.179	45.948
	71.557	59.475	64.462	60.855
	70.773	73.315	69.477	65.747
	74.261	73.505	75.099	65.654
