# Supplementary material for: Capsaicin reduces Alzheimer-associated tau changes in the hippocampus of type 2 diabetes rats
Source: PLoS One. 2017 Feb 22;12(2):e0172477. doi: 10.1371/journal.pone.0172477 (PMC5321461; doi:10.1371/journal.pone.0172477)
Supplement: S3 Table — Fasting blood glucose and fasting plasma insulin levels of rats in different groups on week -12, day -3, day 0 and day 10. (PDF) [file pone.0172477.s004.pdf]

Glucose, mmol/L	-12w	-3d	0d	10d
NC	5.8	4.5	2.8	5.2
	4	3.5	6.7	3.2
	4.4	5.9	8	6.5
	5.6	7.2	3.4	6.9
	6.3	5.7	5.7	3
	5.5	6.6	7.2	4.5
	4.2	3.5	3.2	3.8
	3.9	5.6	7.9	6
	5.7	3.4	6.9	7.7
	6.3	3.6	3.4	4.2
	3.8	3.3	3.2	3.7
NC+CAP	6.3	4.5	6.8	7.3
	6.9	5.9	6.7	3.9
	3.3	6.2	5.4	2.8
	3.8	2.9	3.2	5.6
	4.1	4.1	3	3.2
	5.8	4.2	5.4	5.8
	4.9	6.3	4.7	4.2
	4.2	6.5	6.1	6
	5.8	5.7	4.3	5.2
	6.2	4	2.9	3.8
	7	5.2	3.8	2.8
T2D	4.5	6.5	22.1	22.3
	5.9	4.5	17.8	16.3
	6.7	7.2	17.8	26.5
	3.2	2.6	16.2	20.2
	2.9	5.6	16.1	23.8
	4.3	6.6	16.8	19.7
	5.9	6.2	24.5	21.8
	3.2	5.3	16.7	19.6
	6.7	5	22.1	23.4
	4.8	7.6	17	19.2
T2D+PF	5.6	7.5	23.4	18.2
	7.2	4.5	19.8	21.2
	3.8	6.7	19.9	17.1
	5.4	4.3	16.4	17.2
	6.1	7.8	17.5	16
	7.8	5.8	19.6	17.2
	4.3	5.6	22.1	23.1
	5.3	4.3	19.4	16.4
	4.8	3.1	20	16.8

	3.2	6.9	16.5	16
T2D+CAP	5.1	7.7	21.3	18.3
	7.5	5.8	17.2	12.7
	3.5	6.9	16.3	13.2
	6.2	4.2	24.3	17.6
	5.1	6.3	22.1	13.1
	5.6	2.9	19.9	12.8
	4.1	3.8	17.4	9.8
	4.8	5.6	16.1	13.4
	3.4	6.7	16	12.6
	6	7.8	16.8	11.6
Insulin, nIU/L	-12w	-3d	0d	10d
NC	16.2	9.8	12.3	8.9
	6.7	4.2	8.9	7.1
	14.3	9.2	10.7	9.8
	8.9	13.1	14.2	13.2
	5.1	7.8	11.2	10.2
	7.1	6.5	5.2	5.4
	18.9	10.7	20.9	15.4
	16	19.8	17.8	18.9
	4.5	10.1	7.8	5.4
	6.1	5.8	7.8	7.6
	12.1	10.6	8.2	9.2
NC+CAP	10.8	12.1	19.3	12.1
	6.7	5.4	21.6	17.8
	12.1	10.2	11.2	13.1
	5.6	10.3	8.9	7.9
	16.4	17.2	15.7	6.9
	10.8	18.9	19.8	8.7
	6.7	7.1	10.7	16
	5.4	6.5	7.6	15.6
	13.1	9.8	16.7	16.7
	8.9	15.1	12.1	10.2
	7.2	5.4	13.2	21.5
T2D	11.6	19	72.3	87.4
	5.4	25.6	102.6	128.5
	6.7	22.1	74.3	90.1
	16.7	27.6	112.5	108.7
	11.2	14.3	87.5	67.4
	8.2	12.3	87.3	96.2

	5.6	19.3	72.5	103
	8.7	31	95.6	87.5
	10.2	14.5	78.4	102.5
	12.3	28.7	82.3	95.1
T2D+PF	12.3	32.1	132.1	106.3
	9.1	10.3	90.5	80.6
	10.2	25.6	112	92.1
	8.7	27.1	78.4	65.4
	4.5	18.9	80.2	82.1
	7.6	19.8	95.2	86.7
	8.3	16.7	118.2	72.3
	18.9	25.2	89.5	76.2
	10.2	20.3	101.2	80.1
	12.3	24.3	84.1	67.5
T2D+CAP	6.5	21.9	98.2	47.9
	8.7	17.8	115.3	67.2
	13.2	26.7	90.3	62.1
	17.8	31	92.1	45.3
	10.3	18.9	72.3	57.8
	6.7	16.8	71.2	47.6
	9.9	13.4	85	67.8
	12.1	27.6	100.2	72.1
	12.2	23.5	79.3	56.2
	14.3	32	87	54.2
